# Delayed treatment of basilar thrombosis in a patient with a basilar aneurysm: a case report

**DOI:** 10.1186/1752-1947-2-353

**Published:** 2008-11-18

**Authors:** T Fakhouri, LD McCullough

**Affiliations:** 1 The University of Connecticut Medical School, 263 Farmington Ave, Farmington, CT 06001, USA; 2 Department of Neurology, MC-1840, the University of Connecticut Health Center, 263 Farmington Ave, Farmington, CT 06001, USA

## Abstract

**Introduction:**

Acute occlusion of the basilar artery is a neurological emergency that has a high risk of severe disability and mortality. Delayed thrombolysis or endovascular therapy has been performed with some success in patients who present after 3 hours of symptom onset. Here we present the first case of delayed intra-arterial thrombolysis of a basilar artery thrombosis associated with a large saccular aneurysm.

**Case presentation:**

A 73-year-old Caucasian man with a history of smoking and alcohol abuse presented to the Emergency Department complaining of diplopia and mild slurred speech and who progressed over 12 hours to coma and quadriparesis. He was found to have a large basilar tip aneurysm putting him at high risk for hemorrhage with lytic treatment.

**Conclusion:**

The treatment options for basilar thrombosis are discussed. Aggressive treatment options should be considered despite long durations of clinical symptoms in basilar thrombosis, even in extremely high risk patients.

## Introduction

Stroke is the leading cause of long-term disability in the US. As life expectancies increase, the burden of this disease will continue to grow. The only FDA approved therapy for stroke is intravenous tissue plasminogen activator (tPA) but this agent must be administered within 3 hours of symptom onset. There has been an increasing use of interventional therapies, that is to say, clot retrieval devices and intra-arterial (IA) thrombolytics administration in patients with severe strokes presenting outside the time window for intravenous tPA. Although there are numerous reports in the literature which demonstrate that delayed IA thrombolysis may improve outcome, especially in posterior circulation strokes with stuttering symptoms [1], there has not been a report of the use of this therapy in a patient with an associated basilar tip aneurysm. Here we present a patient who progressed over >12 hours to complete basilar occlusion and quadriparesis who was treated with lytics despite the long duration of symptoms and a large basilar tip aneurysm.

## Case presentation

A 73-year-old Caucasian man with a history of smoking and alcohol abuse presented to the Emergency Department (ED) complaining of the abrupt onset of diplopia and mild slurred speech upon awakening at 8 a.m. He initially thought the symptoms were due to fatigue. Approximately 4 hours after his initial symptoms, he noted minimal right-sided weakness and came to the ED. On admission, the patient was awake and alert but was slightly confused and not oriented to place. Physical examination revealed mild dysarthria, a partial left 3rd cranial nerve palsy with mild abduction of the left eye and a 4 mm pupil that was minimally reactive, with a briskly reactive 2.5 mm pupil on the right. He had a mild right central 7th nerve palsy as well as mild right upper extremity weakness with a pronator drift. Sensory exam was intact to pin prick and touch. Computed tomography (CT) scanning of the head was obtained urgently and showed no hemorrhage or acute ischemic changes. An urgent computed tomography angiogram (CTA) was performed that demonstrated a tortuous tip of the basilar artery with "possible aneurysm vs. clot". A second incidental 5 mm aneurysm was seen in the Middle Cerebral Artery. As the patient was clinically stable, and it was unclear if there was a clot in an underlying basilar aneurysm, he was admitted to the neurological intensive care unit (ICU) for observation. He was placed on aspirin (325 mg) at that time.

**Figure 1:**
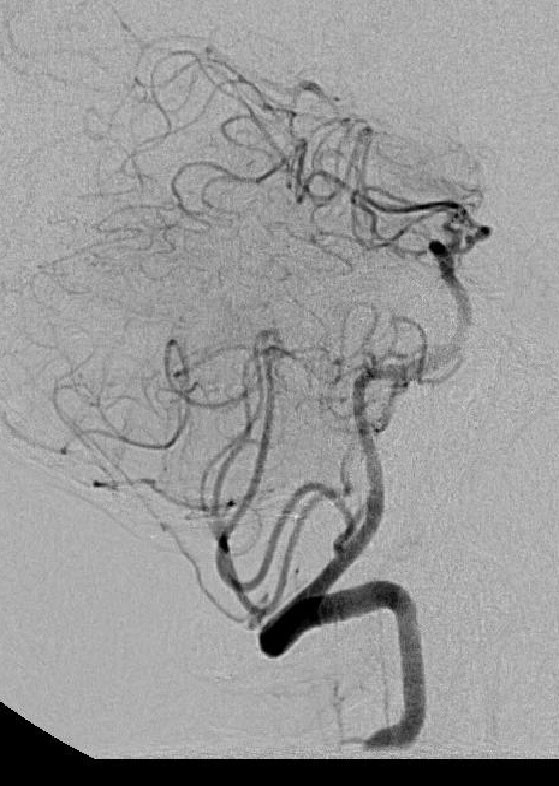
**Cerebral angiogram demonstrating calcified abnormality in the distal basilar artery.** Flow is seen in the distal basilar artery after 10 mg of tissue plasminogen activator was infused in a pulsatile fashion over 30 minutes.

**Figure 2:**
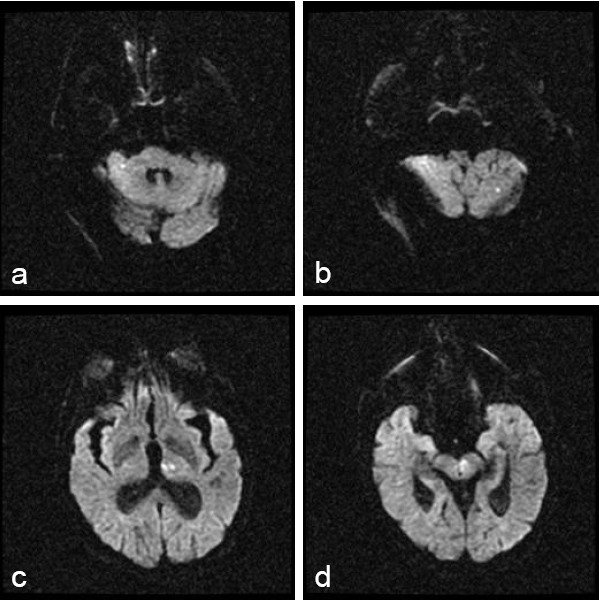
Diffusion-weighted magnetic resonance image showing punctate diffusion bright lesions in the cerebellum (panels a and b), thalamus (c), and midbrain (d).

Over the next 8 hours, the patient became progressively obtunded and developed a 2/5 quadriparesis, a complete left 3rd cranial nerve palsy, and complete right ophthalmoplegia. At approximately 14 hours after the onset of symptoms, he was urgently intubated for airway control, decreasing mental status and loss of gag reflex. He was brought to the endovascular suite for angiography and possible IA tPA for a progressive basilar thrombosis despite the known aneurysm. A near occlusion was found at the basilar tip after the origins of the superior cerebellar arteries, which was associated with a large, complex calcified basilar tip aneurysm, and which was likely a nidus for clot formation. From a position just proximal to the occlusion, 10 mg of tPA was infused in a pulsatile fashion over 30 minutes. Angiography showed improved flow through the distal basilar artery, with no change in the appearance of the calcified dilation of the basilar tip or extravasation of dye (Figure 1). The patient was sent to the ICU. Diffusion magnetic resonance imaging (MRI) the following morning showed multiple tiny diffusion bright lesions in the cerebellum, thalamus and midbrain. This was consistent with an aborted basilar artery occlusion with evidence of ischemia throughout the entire basilar artery vascular territory (Figure 2). On examination the following afternoon, the patient had near complete resolution of symptoms, and was discharged to rehabilitation 9 days later with a mild right 6th cranial nerve palsy. He was discharged on Warfarin with an International Normalized Ratio (INR) of 2.0 as the ischemic changes seen on MRI were minimal, suggesting a low risk of hemorrhagic conversion. No dye extravasation was seen from the aneurysm. It was felt that he was at high risk for recurrent thrombosis. The plan was for non-emergency endovascular coiling of the basilar tip aneurysm 2 months after discharge; however, the patient was lost to follow-up and did not return to the vascular clinic.

## Discussion

Due to a lack of data from randomized clinical trials, administration of IA thrombolysis for patients with basilar artery occlusion remains controversial [2]. Given the poor natural history of the disease and the low (<20%) estimated spontaneous recanalization rate, some neurologists feel that the survival benefit of IA thrombolysis predicted from published case series is sufficient evidence for its use. Among the 10 studies of IA thrombolysis for acute basilar occlusion published in the English literature, there was an aggregate recanalization rate of 64%, and an overall 48% absolute risk reduction for death among patients who recanalized versus those who did not [3]. Without recanalization, the likelihood of good outcome is less than 5% [4]. Reports have varied regarding the effect of delayed administration of IA thrombolysis for basilar occlusion. One case series reported that treatment beyond the 6-hour window resulted in no increased risk of hemorrhage among 20 patients treated for basilar occlusion [1]. A similar study of 26 patients showed no association between survival and the treatment interval [5], while another reported a statistically significant decrease in recanalization rate in patients treated beyond 6 hours of symptom onset [6]. A recent report suggested that even symptomatic chronic basilar occlusions (>80 days) may be improved by vascular intervention [7], representing the ability of this area to survive despite low cerebral blood flow.

For the patient described in this report, delayed IA thrombolysis was pursued as a final measure to reverse a rapid deterioration in his condition despite concerns regarding the structural abnormalities in the distal basilar artery. Post-lysis MRI demonstrated small areas of injury throughout the vascular territory of the basilar artery that in conjunction with his deteriorating clinical exam, suggested that the entire basilar territory (that is to say, thalamus, midbrain and medulla) was at risk for infarct (Figure 2). His declining mental status was likely due to thalamic ischemia. Despite the delay in treatment, we were able to salvage this "at risk" territory and the patient was left with minimal deficits.

## Conclusion

This case illustrates a complex management issue in a patient with basilar thrombosis in the setting of a large basilar tip aneurysm. The possibility that flow changes could occur with aggressive endovascular treatment and reperfusion that could lead to aneurysm rupture needed to be considered in the risk/benefit assessment of treatment. In addition, although it has been well described in the literature that late (>3 hours) treatment of basilar occlusion can lead to good outcomes, and that the natural history without treatment is bleak, there are no large prospective trials showing the benefit of late intervention. Many of these procedures are done outside of the classic "therapeutic window", as was done in this patient due to his rapid clinical deterioration. The risks of these procedures when done outside of a clinical trial must be discussed with the patient (if possible) and family, especially in a high-risk, unusual case such as this. To date, the use of IA thrombolytics in dissecting arterial aneurysms or for thrombosis during aneurysm coiling has been described in the literature [7] but this is the first report of a spontaneous thrombosis in a saccular aneurysm treated with delayed thrombolytics.

## Abbreviations

CTA: computed tomography angiogram; ED: emergency department; IA: intra-arterial; ICU: intensive care unit; MRI: magnetic resonance imaging; Strength testing is listed as 2/5 as a standard neurological score where 5/5 is maximal strength; tPA/Altepase: tissue plasminogen activator

## Consent

Written informed consent was obtained from the patient for publication of this case report and any accompanying images. A copy of the written consent is available for review by the Editor-in-Chief of this journal.

## Competing interests

The authors declare that they have no competing interests.

## Authors' contributions

TF was a major contributor in writing the manuscript and performing the literature review. LDM interpreted the patient data and was a major contributor in writing the manuscript. Both authors read and approved the final manuscript.
